# ICEKAT: an interactive online tool for calculating initial rates from continuous enzyme kinetic traces

**DOI:** 10.1186/s12859-020-3513-y

**Published:** 2020-05-14

**Authors:** Michael D. Olp, Kelsey S. Kalous, Brian C. Smith

**Affiliations:** Department of Biochemistry, Medical College of Wisconsin, 8701 Watertown Plank Rd., Milwaukee, 53226 USA

**Keywords:** Enzyme assay, Enzyme inhibition, Computer program, Michaelis-Menten, Sirtuin

## Abstract

**Background:**

Continuous enzyme kinetic assays are often used in high-throughput applications, as they allow rapid acquisition of large amounts of kinetic data and increased confidence compared to discontinuous assays. However, data analysis is often rate-limiting in high-throughput enzyme assays, as manual inspection and selection of a linear range from individual kinetic traces is cumbersome and prone to user error and bias. Currently available software programs are specialized and designed for the analysis of complex enzymatic models. Despite the widespread use of initial rate determination for processing kinetic data sets, no simple and automated program existed for rapid analysis of initial rates from continuous enzyme kinetic traces.

**Results:**

An Interactive Continuous Enzyme Kinetics Analysis Tool (ICEKAT) was developed for semi-automated calculation of initial rates from continuous enzyme kinetic traces with particular application to the evaluation of Michaelis-Menten and EC_50_/IC_50_ kinetic parameters, as well as the results of high-throughput screening assays. ICEKAT allows users to interactively fit kinetic traces using convenient browser-based selection tools, ameliorating tedious steps involved in defining ranges to fit in general purpose programs like Microsoft Excel and Graphpad Prism, while still maintaining simplicity in determining initial rates. As a test case, we quickly analyzed over 500 continuous enzyme kinetic traces resulting from experimental data on the response of the protein lysine deacetylase SIRT1 to small-molecule activators.

**Conclusions:**

ICEKAT allows simultaneous visualization of individual initial rate fits and the resulting Michaelis-Menten or EC_50_/IC_50_ kinetic model fits, as well as hits from high-throughput screening assays. In addition to serving as a convenient program for practicing enzymologists, ICEKAT is also a useful teaching aid to visually demonstrate in real-time how incorrect initial rate fits can affect calculated Michaelis-Menten or EC_50_/IC_50_ kinetic parameters. For the convenience of the research community, we have made ICEKAT freely available online at https://icekat.herokuapp.com/icekat.

## Background

Continuous enzyme kinetic assays allow rapid acquisition of large amounts of kinetic traces. Therefore, data analysis often becomes the bottleneck of high-throughput enzyme kinetic assays. In cases where IC_50_/EC_50_ values or the Michaelis-Menten parameters *V*_max_ (or *k*_cat_) and *K*_M_ are of principle interest, reduction of kinetic traces to initial rates avoids error arising from assumptions involved in analyzing the entire kinetic trace [1]. The two primary methods for determining initial rates from kinetic traces are *(i)* estimation of the early linear portion of the curve and *(ii)* methods using integrated forms of kinetic equations [2–4]. Currently available programs such as FITSIM [5], DYNAFIT [6], ENZO [7], PCAT [8], and KinTek offer sophisticated routines for fitting kinetic traces. These programs are useful for selecting among complex enzymatic models and analyzing experiments carried out under conditions that may not satisfy the assumptions associated with Michaelis-Menten kinetics [9, 10], for example measuring catalysis inside cells. However, the additional complexity offered by these programs is often not required when analyzing in vitro experiments, making them inefficient and unnecessarily complicated for many continuous enzyme kinetic applications.

In cases where complex kinetic programs are not required, scientists often resort to manual inspection, selection, and fitting of a linear range from each individual kinetic trace using graphing programs such as Microsoft Excel or GraphPad Prism. This approach is time-consuming and susceptible to human error or bias, particularly when low substrate concentrations result in significant curvature of the observed continuous kinetic trace. For our own studies, we unsuccessfully searched for programs that expedited determination of initial rates from continuous enzyme kinetic traces. To fill this void, we developed an Interactive Continuous Enzyme Kinetics Analysis Tool (ICEKAT) for semi-automated initial rate calculations that maintains simplicity while allowing rapid and user-interactive visualization of initial rate fits. For the convenience of the research community, we converted ICEKAT into a publicly-available, browser-based program (https://icekat.herokuapp.com/icekat) to which users can upload series of kinetic traces in comma separated values (CSV) format and download the resulting table of initial rates for further analysis and plotting. ICEKAT has several advantages over other available programs for analyzing enzyme kinetics experiments in that it is free, open source, and does not require any downloads or installations prior to use (Table 1). In addition, ICEKAT includes a plot of the Michaelis-Menten (or IC_50_/EC_50_) fit for the uploaded experiment that is automatically updated based on user interaction with the time ranges used to calculate initial rates (Table 1). As a result, we have found that ICEKAT also serves as a useful teaching aid when demonstrating how incorrect fitting of initial rates from kinetic traces can affect the Michaelis-Menten or IC_50_/EC_50_ parameters calculated from an experiment.

**Table 1:** Comparison of available software programs for fitting kinetic data

**Software**	**Free of charge and open source**	**No downloads required**	**Optimized for interactive analysis of Michaelis-Menten, EC**_**50**_**/IC**_**50**_**, and HTS experiments**
ICEKAT	✓	✓	✓
FITSIM	✓	-	-
DYNAFIT	✓	-	-
ENZO	✓	✓	-
PCAT	✓	-	-
KinTek	-	-	-

## Implementation

### Web-based program for continuous enzyme kinetic analysis

All calculations are carried out in Python using numpy, and both linear and non-linear regression is performed using the Model and curve_fit functions from the lmfit and scipy.optimize modules, respectively. Measured signal can be converted to substrate concentration according to a user-defined transform equation entered into a text box. In linear mode, slopes corresponding to initial rates are determined using a straight line fit to a user-specified segment of the kinetic trace. In logarithmic mode, selected kinetic traces are fit to a logarithmic approximation of the integrated Michaelis-Menten equation defined by

$$y = y_{o} + b \times \ln(1 + t / t_{o})$$

where *y*_o_ is the background signal, *t*_o_ > 0 is the scale of the logarithmic curve, and *b* > 0 is a shape parameter [3]. The kinetic trace slope corresponding to the initial rate is equal to the first derivative of the logarithmic fit when *t* = 0. In Schnell-Mendoza mode, kinetic data is globally fit to the closed form solution to the Michaelis-Menten equation for uncompetitive enzymatic reactions previously described by Schnell and Mendoza [11]

$$[S](t) = K_{M} W \Bigg(\frac{[S_{0}]}{K_{M}} exp \Bigg(\frac{-V_{max}t + [S_{0}]}{K_{M}} \Bigg) \Bigg) $$

where [*S*] is the substrate concentration, *K*_M_ is the Michaelis constant, *W* is the omega function [12] (implemented using the lambertw function from scipy.special), [*S*_0_] is the initial substrate concentration, and *V*_max_ is the maximum reaction velocity. The source code for ICEKAT is freely available at https://github.com/SmithLabMCW/icekat.

## Results and discussion

### Publicly-available webtool for semi-automated and interactive initial rate calculations

**Fig. 1:**
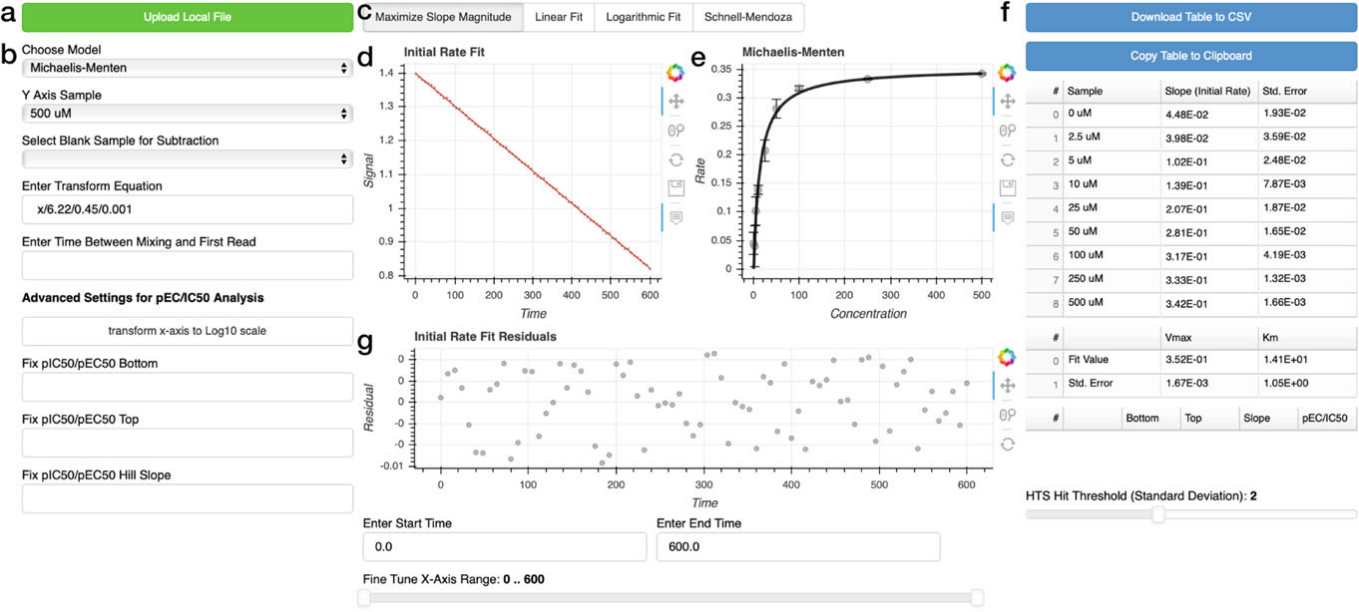
Interactive continuous enzyme kinetic analysis tool. **a** Click "Upload Local File" to begin analysis of user CSV formatted data. **b** Use dropdown menus to select between Michaelis-Menten, IC_50_/EC_50_, and high-throughput screening (HTS) modes, choose y-axis sample, and select a blank sample for subtraction. Use the boxes to "Enter Transform Equation" to transform measured signal into substrate concentration and to enter a time delay between mixing and first read (used in "Logarithmic Fit" mode only). Advanced settings for pIC50/EC50 analysis to transform the input concentration values from a linear to a log scale for analysis and plotting, fix the bottom and/or top of the fitted curve to a particular value, and/or fix the Hill slope of the fitted curve to a particular value (typically 1). **c** Click buttons to select routine for fitting the kinetic traces. The default is to maximize the slope magnitude. **d** Representative continuous enzyme kinetic trace (grey) with initial rate fit (red) corresponding to the selected y-axis sample. **e** Plot of a Michaelis-Menten fit to the calculated initial rates. **f** Data table containing initial rate values and model fit values with errors propagated from the initial rate fits. Use the "Download Table to CSV" or "Copy Table to Clipboard" buttons to export initial rate values from the data table. **g** Plot of the residuals from the kinetic trace initial rate fit in **d**. The "Enter Start Time" and "Enter End Time" boxes and fine tune slider allow the user to optimize the x-axis time domain of the fit to obtain a random residual distribution

Continuous enzyme kinetic traces are uploaded in CSV format using the green button labeled "Upload Local File" at the top of the page (Fig. 1a). While no uploaded data is saved by ICEKAT, users concerned about privacy can download the associated GitHub repository (https://github.com/SmithLabMCW/icekat) and run the application locally. Each CSV file should have one column containing time in seconds or minutes. The remaining CSV columns should contain time-course data, where each column heading contains a number corresponding to titrant concentration (an example CSV file for Michaelis-Menten fitting is included as Appendix C and at https://github.com/SmithLabMCW/icekat/blob/master/icekat/test.csv). Depending on the type of experiment being analyzed, users can choose to fit datasets in Michaelis-Menten, EC_50_/IC_50_, or high-throughput screening (HTS) modes using the dropdown menu labeled "Choose Model" (Fig. 1b). Upon file upload, all kinetic traces are automatically fit to a straight line that maximizes slope magnitude (Fig. 1c), the model fit for the dataset (Fig. 1e) is plotted to the right of the selected trace (Fig. 1d), and the initial rate and model fit values with propagated errors are listed in data tables (Fig. 1f). Users can select individual kinetic traces using the dropdown menu "Y Axis Sample" (Fig. 1b) and manually refit subsets of the time-course data to obtain random residual distributions by entering start and end times in the "Enter Start Time" and "Enter End Time" text boxes and fine tuning the x-axis range using the slider tool (Fig. 1g). Upon refitting an individual kinetic trace, the model fit plot (Fig. 1e) and the data tables (Fig. 1f) are automatically updated. Users may subtract the slope of a blank sample from the rest of the dataset using the "Select Blank Sample for Subtraction" dropdown menu (Fig. 1b). Users can also transform measured signal into meaningful substrate concentrations by entering a transform equation (signal as a function of time "x", e.g. "x/*(extinction coefficient × path length × enzyme concentration)*") in the "Enter Transform Equation" box (Fig. 1b). Finally, the initial rates listed in the table at the right can be copied to the clipboard by clicking the blue button labeled "Copy Table to Clipboard" or downloaded as a CSV file using the blue button labeled "Download Table to CSV" (Fig. 1f). To encourage wide adoption of ICEKAT, we have created a tutorial (Appendix B).

### Kinetic trace fitting routines

The most common methods for determining initial rates from continuous enzyme kinetic traces are *(i)* estimation of the early linear portion of the trace and *(ii)* methods using integrated forms of kinetic equations [2–4]. These integrated kinetic equations are particularly important when the portion of the kinetic trace corresponding to the initial rate is difficult to measure, as in situations where substrate concentrations are below the *K*_M_ value of an enzyme. Early methods using the integrated Michaelis-Menten equation were sensitive to assumptions regarding reaction reversibility, product inhibition, and enzyme inactivation and stability [1]. More recent methods, which treat initial and final substrate concentrations as parameters in non-linear regression [2–4], eliminate these assumptions from the fitting process and have greatly increased the applicability of the integrated Michaelis-Menten equation to calculating initial rates from kinetic traces. ICEKAT provides the user flexibility in the method used to determine initial rates.

**Fig. 2:**
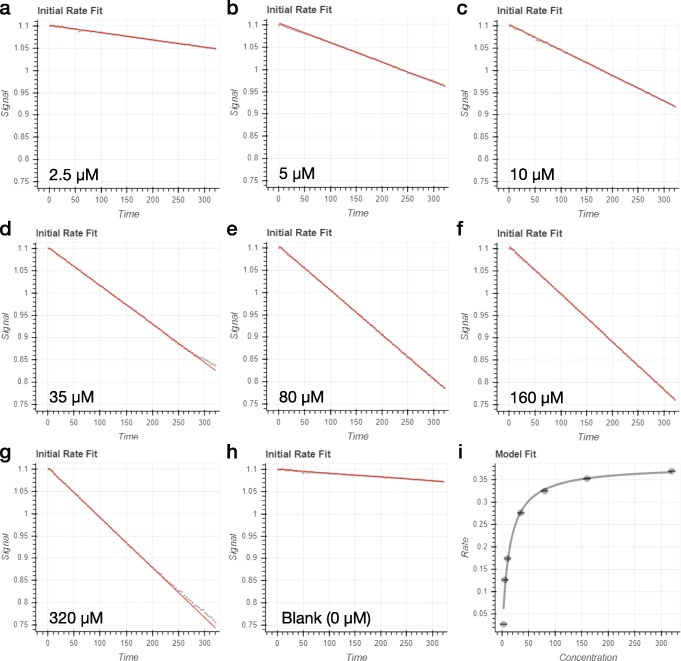
Automated determination of steady-state kinetic parameters. **a-h** Automated fits (red lines) generated by ICEKAT from a representative dataset using substrate concentrations ranging from 0 to 320 *μ*M (grey points). **i** Michaelis-Menten plot automatically generated by ICEKAT

ICEKAT first generates an initial rate prediction using linear regression to maximize the first derivative of the kinetic traces smoothed by cubic spline interpolation. Using this method, linear initial rate estimations are automatically generated for an entire experiment (e.g. substrate titration to generate a Michaelis-Menten plot; Fig. 2a-h). To avoid error arising from erroneous fitting of kinetic artifacts by the program, ICEKAT allows interactive re-assignment of the time range used for fitting by entering start and end times in the "Enter Start Time" and "Enter End Time" boxes and then fine tuning the x-axis range using the slider tool (Fig. 1g). Upon manual selection of a new time range, a new initial rate is calculated, and this change is automatically reflected in the overall kinetic model fit (Fig. 1e) and data tables (Fig. 1f). During the process of fitting initial rates, the user can select from four fitting modes by clicking on the buttons labeled "Maximize Slope Magnitude", "Linear Fit", "Logarithmic Fit", and "Schnell-Mendoza" ("Schnell-Mendoza" is only available in Michaelis-Menten fitting) (Fig. 1c). "Maximize Slope Magnitude" mode is the default and is used in the automatic initial rate estimation described above. "Linear Fit" mode is equivalent to fitting a straight line to the user-selected portion of the kinetic trace. "Logarithmic Fit" mode is an implementation of the logarithmic approximation of the integrated Michaelis-Menten equation, as described by Lu and coworkers [3]. The "Logarithmic Fit" mode is particularly useful to avoid under-estimation of initial rates from kinetic traces where an initial linear segment cannot be satisfactorily identified. If there is a significant time delay between initiating the enzyme-catalyzed reaction and the first sample read, a time value can be entered into the text box labeled "Enter Time Between Mixing and First Read" (Fig. 1b) to extrapolate initial rate calculations back to the exact time of mixing (note, the value entered in this text box is only used in the calculation when "Logarithmic Fit" is selected). Regardless of the fitting method used, the errors from the initial rate fitting are propagated into fitting to the Michaelis-Menten (or IC_50_/EC_50_) equation. Throughout the fitting process, users should strive to obtain a random distribution of points in the kinetic trace fit residual plot located directly below the kinetic trace (Fig. 1g). In addition, users can dynamically assess how changes in initial rate calculations for each kinetic trace affect the overall fit of a titration to the Michaelis-Menten (or IC_50_/EC_50_) equation. However, extreme caution must be observed to avoid manipulating the fitted time ranges solely to provide a "better" fit to the Michaelis-Menten equation.

It is crucial to note that each method of estimating initial rates is associated with its own limitations. For instance, analysis of the linear portions of enzyme-substrate reactions carried out under low substrate concentrations can lead to underestimated initial rate determinations. Conversely, similar underestimation of initial rates can occur with forms of the integrated Michaelis-Menten equation when high substrate concentrations are not sufficiently depleted during the experimental measurement [2–4]. As a result, we have also included the closed form solution described by Schnell and Mendoza [11] which describes both the initial and subsequent quasi-steady-state phases of non-competitive enzyme-substrate reactions. In "Schnell-Mendoza" mode (Michaelis-Menten fitting only) (Fig. 1c), *K*_M_ and *V*_max_ values are determined using a global fit to all available kinetic traces (Figure S1). In this context, the "Select Blank Sample for Subtraction" option subtracts a straight line derived from the selected trace from the dataset. Unlike the other modes, in "Schnell-Mendoza" mode the data must be plotted as a decrease in substrate concentration over time. The "Enter Transform Equation" text box (Fig. 1b) allows conversion between measured signal to substrate concentrations (note this transform may need to be inverted through multiplying by -1 when analyzing experiments that measure increased product concentration over time). It is important to note that the Schnell-Mendoza closed form solution is not an ’exact’ solution and (similar to all enzyme kinetics experiments that approximate a quasi-steady-state) should only be used for experimental conditions where [*E*_0_]/(*K*_M_ + [*S*_0_]) ≪ 1 (where [*E*_0_] is the initial enzyme concentration and [*S*_0_] is the initial substrate concentration) [11].

### EC_50_/IC_50_ and high-throughput screening modes

**Fig. 3:**
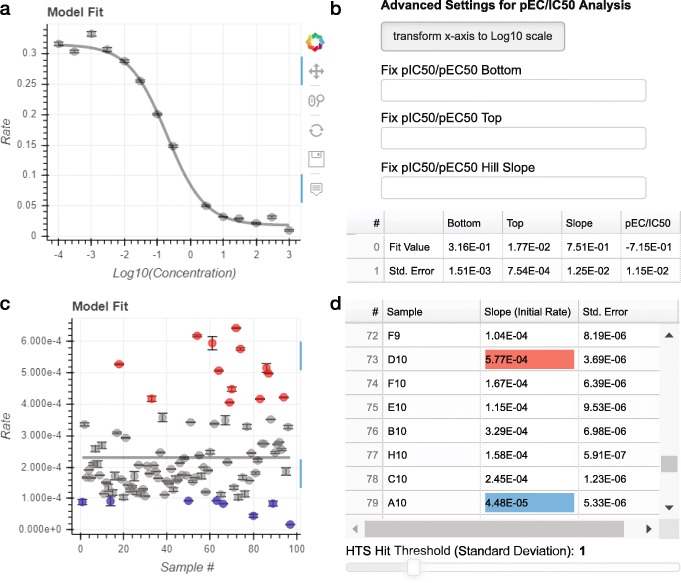
EC_50_/IC_50_ and high-throughput screening modes. **a** Plot of a representative IC_50_ model fit of initial rates. **b** Widgets for choosing advanced EC_50_/IC_50_ analysis settings allow users to convert the x-axis to Log_10_ scale and fix regression parameters. The data table displays fit values with errors propagated from the initial rate fits for the 4-parameter logistic model. **c** Plot displaying HTS analysis of initial rates from a representative 96-well plate. **d** The data table displays initial rates and associated errors for all samples uploaded and highlights cells corresponding to samples with initial rates above (red) or below (blue) the standard deviation threshold defined by the slider (here set to 1 standard deviation from the mean initial rate)

In addition to Michaelis-Menten kinetics, ICEKAT is optimized to perform analysis of datasets resulting from EC_50_/IC_50_ (Fig. 3a/b) and HTS (Fig. 3c/d) kinetic experiments, defined in the "Choose Model" dropdown menu (Fig. 1b). Example CSV files for EC_50_/IC_50_ fitting and high-throughput screening analyses are included as Appendix D and Appendix E. In each case, initial rates are determined in the same manner as described above. When working in EC_50_/IC_50_ mode, changes in initial rate values and associated errors are automatically reflected in the fit to the 4-parameter logistic model (Fig. 3a):

$$y = bottom + \frac{top-bottom}{1+{10}^{Hill Slope \times (pIC^{50}-x)}}$$

Advanced EC_50_/IC_50_ analysis settings allow users to inter-convert the x-axis between linear and Log_10_ scale, as well as fix the top, bottom, and Hill slope regression values (Fig. 3b). The data table containing the four regression parameters and propagated errors is automatically updated throughout interactive initial rate fitting (Fig. 3b). It is important to note that IC_50_ values such as those provided by ICEKAT are empirical and highly dependent on experimental conditions (*i.e.* substrate concentrations) as well as the mechanism of inhibition (competitive/uncompetitive/noncompetitive, reversible/irreversible/tight binding, cooperative/allosteric, etc.). As a result, we encourage users interested in converting IC_50_ values to fundamentally-based *K*_i_ values to use the Cheng Prussoff equations [13].

In HTS mode, an unlimited number of samples (e.g. activator/inhibitor screening in 96- or 384-well plate format) can be uploaded and fit to determine initial rates. When analyzing data in HTS mode, a straight horizontal line is plotted to represent the mean initial rate of the data set, and samples associated with initial rates either above (red) or below (blue) a user-defined standard deviation threshold from the mean are highlighted on the model fit plot (Fig. 3c) and in the data table (Fig. 3d).

### Case-study: interactive dataset fitting as a visual teaching aid

**Fig. 4:**
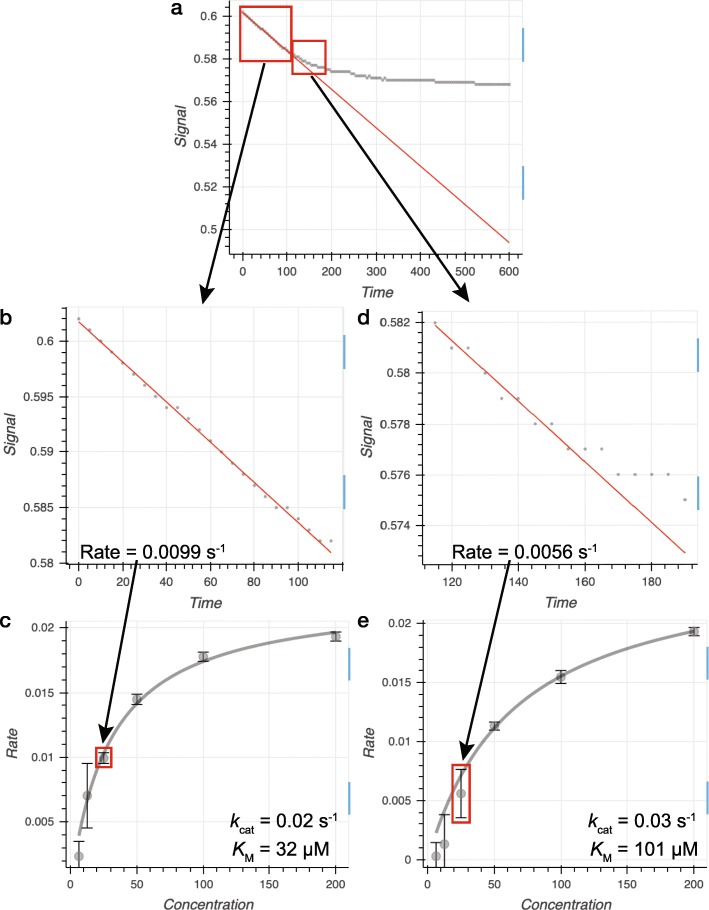
Interactive dataset fitting as a visual teaching aid. **a** A representative continuous enzyme kinetic trace where either **b** the initial linear rate is fit appropriately yielding **c** initial rates for the Michaelis-Menten fit or **d** the kinetic trace is fit after the initial rate time region has passed yielding **e** a Michaelis-Menten fit with a *K*_M_ value higher than the actual *K*_M_ value

We have found that a key use of ICEKAT is to teach students or train new laboratory members in fitting continuous enzyme kinetic data. In particular, ICEKAT can be used to interactively demonstrate proper identification and selection of the initial rate component of a kinetic trace, as well as the consequences of incorrect identification of initial rates (Fig. 4). Fitting a segment of a kinetic trace temporally downstream of the initial rate segment results in underestimation of the initial rate (Fig. 4a/d/e). In Michaelis-Menten analysis, underestimation of rates, especially from kinetic traces where the concentration of substrate is low, can result in either overestimation of the *K*_M_ value for the enzyme (Fig. 4c/e) or give rise to sigmoidal rather than hyperbolic kinetics that wrongly suggest allostery [14]. This phenomenon can be demonstrated by using the "Enter Start Time" and "Enter End Time" boxes (Fig. 1g) to intentionally select an incorrect line segment after the initial rate component from continuous enzyme kinetic data. As ICEKAT automatically updates the overall fit of the entire data set (*K*_M_ and *V*_max_ or *k*_cat_ values) as adjustments are made, students and trainees are able to immediately visualize the impact of underestimating an initial rate on the overall fit of a Michaelis-Menten curve (Fig. 4d/e). Adjustment of the "Enter Start Time" and "Enter End Time" boxes to fit different components of a curve, and rapid integration of the adjusted rates into the overall fit, allows fluid demonstration of initial rate fitting in the context of a lecture in real time, which otherwise would be discontinuous and cumbersome using programs such as Microsoft Excel or GraphPad Prism.

### Case-study: rapid determination of initial rates and steady-state kinetic parameters for SIRT1 variants with small-molecule activators

The SIRT1 deacetylase [15] protects against aging-related diseases [16–18], and SIRT1 activators (STACs) [19–23] are sought as therapeutics. Resveratrol and other STACs (Figure S2A) activate SIRT1 by lowering the *K*_M_ value towards a subset of acetylated substrates [19, 20, 22]. However, the relative importance of *N*-terminal domain (residues 183–230) versus catalytic core residues (residues 244–498) in SIRT1 activation was unknown.

To test the ability of ICEKAT to rapidly determine steady-state kinetic parameters from continuous enzyme kinetic traces, six SIRT1 variants (I223A, I223R, E230K, D292A, F414A, and R446E) were generated based on previous structural and kinetic studies [19, 21, 22] using site-directed mutagenesis (Table S1). Each variant was screened in the presence or absence of resveratrol or STAC1 (Figure S2A) using a continuous enzyme-coupled assay for sirtuins [24]. Given the combinatorial nature of this study (seven SIRT1 variants, seven substrate concentrations, two STACs, and at least three replicates), over 500 kinetic traces were generated, which provided an excellent test case of ICEKAT for semi-automated processing of steady-state kinetic data. The kinetic parameters *k*_cat_ (Figure S2b), *K*_M_ (Figure S2c), and *k*_cat_/*K*_M_ (Figure S2d) were calculated to determine the impact of each variant on SIRT1 activation. Our data indicate that I223, D292, F414 and R446 are required for both resveratrol- and STAC1-mediated SIRT1 activation. Interestingly, the E230K variant was selectively activated by STAC1, indicating the SIRT1 binding site and/or activation mechanism are not identical for all sirtuin activating compounds (see Appendix A for additional Discussion). In our experience, analysis of kinetic traces, such as from this case-study, is accomplished at least 3 times faster using ICEKAT relative to processing via Microsoft Excel.

## Conclusions

To increase speed at the data analysis stage of continuous enzyme kinetic assays, a publicly available, web-based program (ICEKAT) was developed for semi-automated and interactive continuous enzyme kinetic trace analysis. ICEKAT offers several advantages over other available programs for analyzing continuous enzyme kinetics experiments in that it is free, web-based, and optimized for interactive and intuitive analysis of Michaelis-Menten, EC_50_/IC_50_, and HTS datasets. As a case study for ICEKAT, a comprehensive kinetic screen using a continuous enzyme-coupled assay for sirtuins [24] was conducted. In addition to increasing the efficiency of continuous enzyme kinetic trace analyses, the interactive nature of the program provides a useful teaching aid to demonstrate the link between initial rate determination and calculation of Michaelis-Menten and EC_50_/IC_50_ parameters. Following the Standards for Reporting Enzymology Data (STRENDA) guidelines for reporting enzyme kinetics data and physical constants [25], depositing fitted kinetics constants in STRENDA DB (https://www.beilstein-strenda-db.org/strenda/index.xhtml), and consistent use of data analysis tools such as ICEKAT, will greatly increase the reproducibility of enzyme assays.

## Availability and requirements

**Project name:** Interactive Continuous Enzyme Kinetics Analysis Tool (ICEKAT)**Project home page:**https://icekat.herokuapp.com/icekat**Archived version:** N/A**Operating system(s):** Platform independent**Programming language:** Python, Java**Other requirements:** N/A**License:** N/A**Any restrictions to use by non-academics:** N/A

## Supplementary information

**Additional file 1** Supplemental materials and methods, discussion, and references. Table S1 (SIRT1 mutagenesis primers), Figure S1 (Calculation of steady-kinetic parameters using the Schnell-Mendoza equation), Figure S2 (SIRT1 variant *k*_cat_ and *K*_M_ values varying acetylated peptide in the presence of resveratrol and STAC1).

**Additional file 2** PDF tutorial for using ICEKAT.

**Additional file 3** Sample continuous kinetic trace input data file for fitting to the Michaelis-Menten equation.

**Additional file 4** Sample continuous kinetic trace input data file for fitting to the iC_50_/EC_50_ equation.

**Additional file 5** Sample continuous kinetic trace input data file for analyzing high-throughput screening data.

## Data Availability

The program described here is freely available at https://icekat.herokuapp.com/icekat. All source code is present in the associated GitHub repository located at https://github.com/SmithLabMCW/icekat. The fitted kinetic constants for the SIRT1 activator case-study are available at STRENDA DB (STRENDA Registry Number: NC2FY0).

## Abbreviations

CSV: Comma separated values
HTS: High-throughput screening
ICEKAT: Interactive continuous enzyme kinetics analysis tool
STACs: Sirtuin activating compounds
