# Supplementary material for: ICEKAT: an interactive online tool for calculating initial rates from continuous enzyme kinetic traces
Source: BMC Bioinformatics. 2020 May 14;21:186. doi: 10.1186/s12859-020-3513-y (PMC7222511; doi:10.1186/s12859-020-3513-y)
Supplement: Supplementary file 1 — Additional file 1 Supplemental materials and methods, discussion, and references. Table S1 (SIRT1 mutagenesis primers), Figure S1 (Calculation of steady-kinetic parameters using the Schnell-Mendoza equation), Figure S2 (SIRT1 variant kcat and KM values varying acetylated peptide in the presence of resveratrol and STAC1). [file 12859_2020_3513_MOESM1_ESM.pdf]

ICEKAT: An interactive online tool for calculating initial rates from continuous enzyme kinetic traces

Michael D. Olp, Kelsey S. Kalous, Brian C. Smith
Department of Biochemistry, Medical College of Wisconsin

Appendix A

Supplemental Materials & Methods

Materials. Resveratrol, (L)-glutamic dehydrogenase (from bovine liver), nicotinamide adenine dinucleotide oxidized form (NAD^+), and trifluoroacetic acid (TFA) were purchased from Sigma-Aldrich (Milwaukee, WI). Fmoc amino acids, α-ketoglutaric acid, and NADH were purchased from Chem-Impex (Wood Dale, IL). Rink-amide 4-methylbenzhydrylamine resin was purchased from Novabiochem. Ni-NTA superflow resin was purchased from 5 PRIME (Hilden, Germany). STAC1 was generous gift from GlaxoSmithKline.

Solid-phase peptide synthesis. A 5-mer acetyl-lysine peptide based on p53 (p53Wac: H_2N-RHKK(acetyl)W-$CONH_2$) was synthesized using tBu/Fmoc solid-phase peptide synthesis on an Applied Biosystems ABI 433A peptide synthesizer (FastMoc 0.1 mmol). Fmoc amino acids were coupled to 100-200-mesh Rink-amide 4-methylbenzhydrylamine resin. Protecting groups used were: boc for lysine and tryptophan, trityl for histidine, and 2,2,4,6,7-pentamethyldihydrobenzofuran-5-sulfonyl for arginine. Amino acids were coupled using 10 equiv of activated amino acid, 9 equiv of 2-(1H-benzotriazol-1-yl)-1,1,3,3-tetramethylluronium hexafluorophosphate, 9 equiv of hydroxybenzotriazole, and 20 equiv of *N,N*-diisopropylethylamine. Following completion of the synthesis, resin was rinsed with dichloromethane and dried. Peptide was then deprotected and cleaved from the resin using 95% (v/v) TFA, 2.5% (v/v) H_2O, and 2.5% (v/v) triisopropylsilane. Peptide was recovered by precipitation with cold (-20 °C) diethyl ether and centrifugation at 4,200 × *g*. Pelleted peptide was washed twice by resuspension in cold diethyl ether and subsequent centrifugation. Precipitated peptide was dried, redissolved in H_2O, frozen in liquid N_2, and lyophilized. Crude peptide was purified via semipreparative reversed phase HPLC on a μBondapak C18 column (Waters, 3.9 × 300 mm) using an Agilent 1100 series HPLC. Peptide was eluted using a gradient of 0-80% v/v acetonitrile in water with 0.1% v/v TFA. Collected fractions were frozen in liquid N_2, lyophilized, and the masses of final products confirmed via direct injection electrospray ionization-mass spectrometry (QExactive, Thermo Scientific). The observed mass matched the predicted mass for the p53Wac peptide (calculated for $C_{39}H_{61}N_{14}O_7$ $[M+H]^+$: 837.48422, found: 837.48619). Peptide stocks were made in ddH_2O and concentrations determined spectrophotometrically using the extinction coefficient for tryptophan (5.6 $mM^{-1}cm^{-1}$).

Site-directed mutagenesis. Plasmids coding for SIRT1 I223A, I223R, E230K, D292A, F414A, and R446E were generated via site-directed mutagenesis of a pET28a-LIC plasmid coding for a truncated wild type human (NCBI Taxonomy ID: 9606) SIRT1 (UniProtKB: Q96EB6; EC: 2.3.1.286; amino acids 156-664) with a *C*-terminal His_6 tag. Mutagenesis primers were purchased from Integrated DNA Technologies (Coralville, IA) (Table S1). Variants were confirmed by DNA sequencing (Retrogen, San Diego, CA).

Expression and purification of SIRT1. Human SIRT1 WT and variants (I223A, I223R, E230K, D292A, F414A, and R446E) were expressed and purified from BL21(DE3) *E. coli* via nickel affinity chromatography as previously described[1]. Briefly, cells were transformed and grown at 37 °C in 2XYT media supplemented with 50 mg/L kanamycin to an optical density of 0.7 at 600 nm. Protein expression was induced with 0.5 mM IPTG at 16 °C overnight. Cells were harvested via centrifugation at 5,000 × *g* and bacterial pellets frozen at -80 °C. Pellets were thawed on ice and resuspended in 20 mM Tris-HCl buffer pH 8.0 at 4 °C containing 500 mM NaCl, 10% v/v glycerol, 5.0 mM β-mercaptoethanol, and 2.5 mM imidazole. Cells were lysed via sonication and insoluble debris cleared via centrifugation at 30,000 × *g*. Ni-NTA resin was added to cleared lysate (0.75 mL Ni-NTA resin/L culture) and rocked at 4 °C for 1 h. Bound resin was pelleted via centrifugation at 4,200 × *g*, resuspended in 10× resin volume of lysis buffer, and applied to a column. Resin was washed with 10× resin volume of 20 mM Tris-HCl buffer pH 8 at 4 °C containing 500 mM NaCl, 10% (v/v) glycerol, 5.0 mM β-mercaptoethanol, and 25 mM imidazole. Purified SIRT1 was eluted with 5× resin volume of 20 mM Tris-HCl buffer pH 8 at 4 °C containing 500 mM NaCl, 10% v/v glycerol, 5.0 mM β-mercaptoethanol, and 300 mM imidazole. SIRT1

was further purified via size-exclusion chromatography using an ENrich SEC 650 10 × 300 mm column, eluting into 10 mM HEPES-NaOH buffer pH 7.5 at 25 °C containing 150 mM NaCl, 10% v/v glycerol, and 1.0 mM DTT. Purity was assessed by SDS-PAGE. Purified SIRT1 was concentrated to >47 μM as determined by Bradford assay using BSA as the standard, aliquoted, flash frozen in liquid N_2, and stored at -80 °C.

Expression and purification of nicotinamidase. *S. enterica* nicotinamidase with an *N*-terminal maltose-binding protein-hexahistidine (MBP-H_6) tag (MBP-PncA) (EC:3.5.1.19) in the pTEV6 plasmid was expressed and purified from BL21(DE3) *E. coli* via nickel affinity chromatography as previously described[2]. Briefly, cells were transformed and grown at 37 °C in 2XYT media supplemented with 50 mg/L ampicillin to an optical density of 0.7 at 600 nm. Protein expression was induced with 0.5 mM IPTG at 25 °C overnight. Cells were harvested via centrifugation at 5,000 × *g* and bacterial pellets frozen at -80 °C. Pellets were thawed on ice and resuspended in 20 mM potassium phosphate buffer pH 7.5 at 25 °C containing 500 mM NaCl and 5 mM imidazole. Cells were lysed via sonication and insoluble debris cleared via centrifugation at 30,000 × *g*. Ni-NTA resin was added to cleared lysate (0.75 mL Ni-NTA resin/L culture) and rocked at 4 °C for 1 h. Bound resin was pelleted via centrifugation at 4,200 × *g*, resuspended in 10× resin volume of lysis buffer, and applied to a column. Resin was washed with 10× resin volume of 20 mM potassium phosphate buffer pH 7.5 at 25 °C containing 500 mM NaCl and 25 mM imidazole. Purified MBP-PncA was eluted with 5× resin volume of 20 mM potassium phosphate buffer pH 7.5 at 25 °C containing 500 mM NaCl and 300 mM imidazole. MBP-PncA was further purified via size-exclusion chromatography using an ENrich SEC 650 10 × 300 mm column, eluting into buffer containing 50 mM potassium phosphate buffer pH 7.5 at 25 °C, 100 mM NaCl, and 10% v/v glycerol. Purity was assessed by SDS-PAGE. Purified MBP-PncA was concentrated to >540 μM as determined by Bradford assay using BSA as the standard, aliquoted, flash frozen in liquid N_2, and stored at -80 °C.

Sirtuin enzyme coupled assay. Activity of SIRT1 was monitored under initial rate conditions (determined from the linear range at <10% conversion of the p53Wac peptide) using a continuous enzyme-coupled microplate assay for sirtuins[2]. The assay was performed at 25 °C in a 150 μL reaction mixture containing 20 mM potassium phosphate pH 7.5 at 25 °C (measured from a 100 mM stock using a pH electrode before adding to the rest of reaction mixture), 2.0 mM NAD^+, 3.3 mM α-ketoglutarate, 200 μM NADH, 2.0 μM MBP-PncA (thawed on ice), 2.5 units of (L)-glutamic dehydrogenase (stored at 4 °C), 0.5 or 1.0 μM SIRT1 WT or variants (thawed on ice), and 6.25-200 μM p53Wac peptide. Nicotinamide formation coupled to NADH consumption was continuously monitored for 10 min following the decrease in absorbance at 340 nm in a 96-well clear flat bottom plate (Greiner Bio-One) using a BioTek Synergy Mx microplate reader (Winooski, VT). To assess SIRT1 activation by resveratrol and STAC1, Michaelis-Menten titrations of p53Wac peptide were performed under saturating concentrations of NAD^+ (2.0 mM), in the presence and absence of saturating concentrations of resveratrol (50 or 100 μM) or 50 μM STAC1. Reactions were initiated via addition of NAD^+. Continuous enzyme kinetic traces were processed using ICEKAT to determine k_{cat}, K_M, and k_{cat}/K_M values. The fitted kinetic constants were deposited in STRENDA DB (doi: 10.22011/strenda_db.NC2FY0).

Table S1. SIRT1 mutagenesis primers

Construct	Forward primer	Reverse primer
I223A	5' GATGATATGACACTGTGGCAGGCTGTTATTAATATCC 3'	5' GGATATTAATAACAGCCTGCCACAGTGTCATATCATC 3'
I223R	5' GATGATATGACACTGTGGCAGCGTGTTATTAATATCC 3'	5' GGATATTAATAACACGCTGCCACAGTGTCATATCATC 3'
E230K	5' GGCAGATTGTTATTAATATCCTTTCAAAACCACC 3'	5' GGTGGTTTTGAAAGGATATTAATAACAATCTGCC 3'
D292A	5' CCCAGATCTTCCAGCTCCTCAAGCGATGTTTG 3'	5' CAAACATCGCTTGAGGAGCTGGAAGATCTGGG 3'
F414A	5' CCAGAGATTGTGTTTGCTGGTGAAAATTTACCAGAAC 3'	5' GTTCTGGTAAATTTTCACCAGCAAACACAATCTCTGG 3'
R446E	5' GGGTCTTCCCTCAAAGTAGAACCAGTAGCACTAATTCC 3'	5' GGAATTAGTGCTACTGGTTCTACTTTGAGGGAAGACCC 3'

Supplemental Discussion

Consistent with previously-published data, wild type SIRT1 was activated by resveratrol and STAC1 (Figure S2a). Although not reaching statistical significance, the K_M value for the p53Wac peptide (14 ± 5 µM) was reduced approximately 3.5-fold by resveratrol (4 ± 1 µM) and approximately two-fold (7 ± 3 µM) by STAC1 (Figure S2d) without perturbation of k_{cat} (Figure S2c). The overall catalytic efficiency (k_{cat}/K_M) of SIRT1 WT (8,662 ± 2,264 $M^{-1}s^{-1}$) was significantly enhanced in the presence of resveratrol (23,273 ± 3,728 M^{-1} s^{-1}) and STAC1 (15,872 ± 3,315 $M^{-1}s^{-1}$) (Figure S2b). k_{cat} values for SIRT1 variants were not significantly enhanced (Figure S2c). p53Wac peptide K_M values for SIRT1 variants were not significantly reduced, with the exception of SIRT1 D292A, which displayed significant reduction in K_M by resveratrol (23 ± 9 µM), relative to the untreated enzyme (62 ± 9 µM) (Figure S2d). Examination of the overall catalytic efficiency of each variant in the presence and absence of resveratrol and STAC1 revealed robust activation of wild type SIRT1, but impaired activation of all variants, with the exception of E230K, which was significantly activated by STAC1 (20,684 ± 3,894 $M^{-1}s^{-1}$) relative to the untreated enzyme (5,514 ± 1,612 $M^{-1}s^{-1}$) (Figure S2b). These data are consistent with a critical role for each tested residue in mediating SIRT1 activation, either via formation of critical intramolecular, SIRT1-STAC, or SIRT1-substrate contacts. However, the differential activation of the E230K variant relative to other SIRT1 mutants suggests the SIRT1 binding site and/or activation mechanism are not identical for all sirtuin activating compounds.

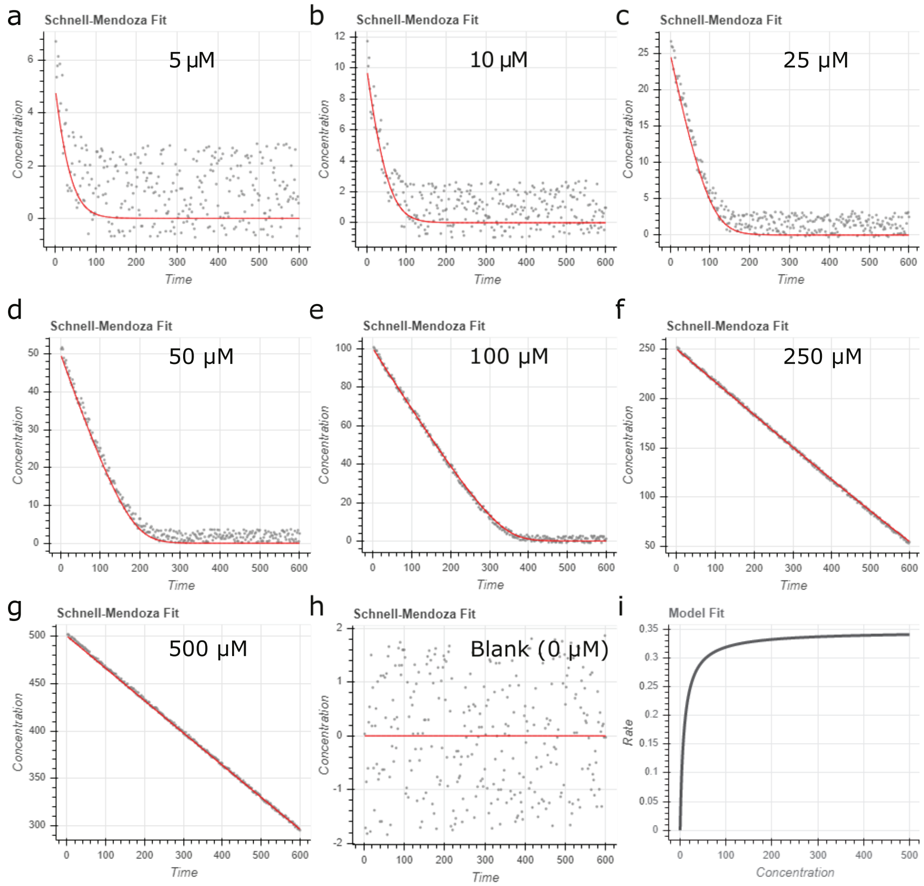

Figure S1. Calculation of steady-kinetic parameters using the Schnell-Mendoza equation[3]. **(a-h)** Global fit (red lines) generated by ICEKAT from a representative dataset using substrate concentrations ranging from 0 to 500 μM (grey points). **(i)** Michaelis-Menten plot automatically generated by ICEKAT.

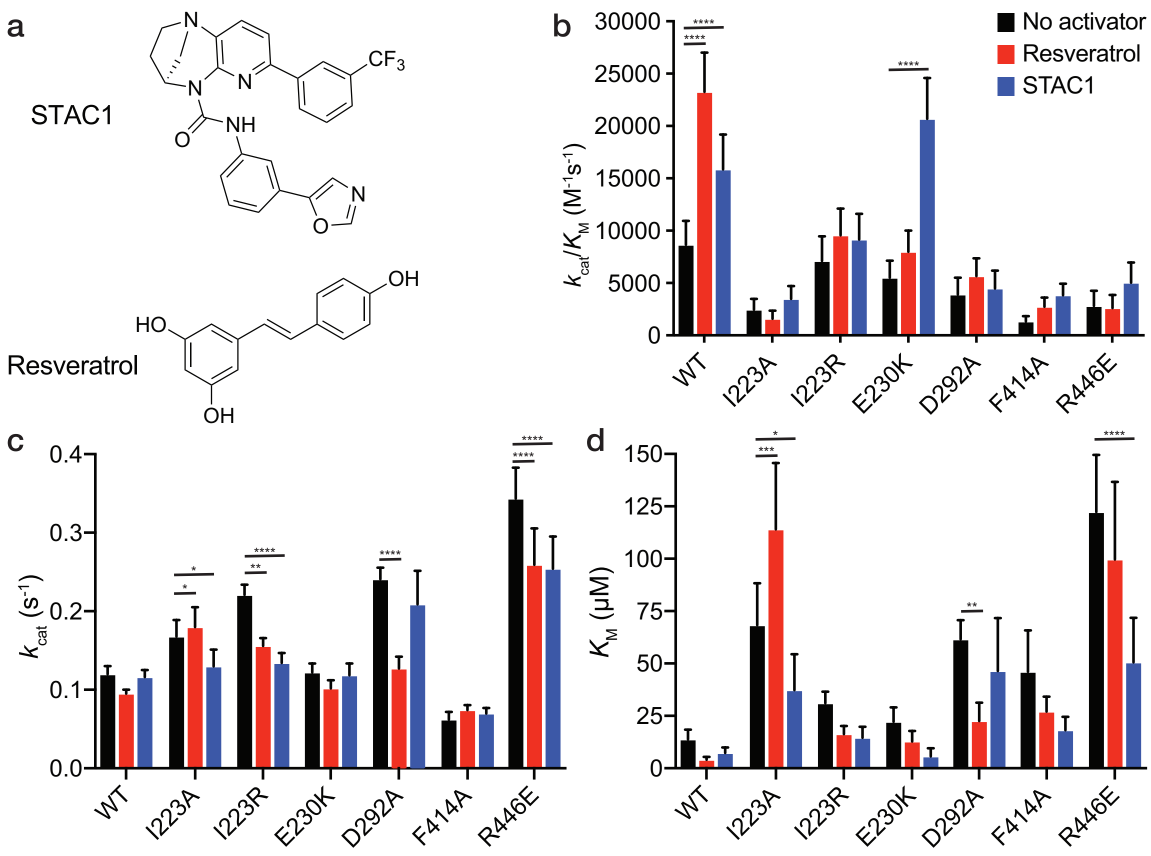

Figure S2. SIRT1 variant k_{cat} and K_M values varying acetylated peptide in the presence of resveratrol and STAC1. (a) Chemical structures of the SIRT1 activating compounds used in this study: resveratrol and STAC1. **(b-d)** p53Wac peptide titrations were performed for SIRT1 WT and SIRT1 variants (I223A, I223R, E230K, D292A, F414A, R446E) (0.50 or 1.0 μM) under saturating concentrations of NAD^+ (2.0 mM). Initial rates were fit using ICEKAT and plotted versus substrate concentration. The **(b)** catalytic efficiency (k_{cat}/K_M), **(c)** turnover number (k_{cat}), and **(d)** the Michaelis constant (K_M) were calculated for each variant in the presence and absence of resveratrol and STAC1. Differences in kinetic parameters were compared via two-way ANOVA (n ≥ 3; plotted as mean ± standard deviation) (* = $p < 0.05$, ** = $p < 0.001$, *** = $p < 0.0005$, **** = $p < 0.0001$). The fitted kinetic constants were deposited in STRENDA DB (doi: 10.22011/strenda_db.NC2FY0).

Supplemental References

1. Kalous, K. S., Wynia-Smith, S. L., Olp, M. D. & Smith, B. C. Mechanism of SIRT1 NAD^+-dependent Protein Deacetylase Inhibition by Cysteine S-Nitrosation. *J Biol Chem* **291**, 25398-25410, doi:10.1074/jbc.M116.754655 (2016).
2. Smith, B. C., Hallows, W. C. & Denu, J. M. A continuous microplate assay for sirtuins and nicotinamide-producing enzymes. *Anal Biochem* **394**, 101-109, doi:10.1016/j.ab.2009.07.019 (2009).
3. Schnell, S., Mendoza, C.: Closed form solution for time-dependent enzyme kinetics. *Journal of Theoretical Biology* **187**(2), 2017-212 (1997). doi:10.1006/jtbi.1997.0425
