# Supplementary material for: ICEKAT: an interactive online tool for calculating initial rates from continuous enzyme kinetic traces
Source: BMC Bioinformatics. 2020 May 14;21:186. doi: 10.1186/s12859-020-3513-y (PMC7222511; doi:10.1186/s12859-020-3513-y)
Supplement: Supplementary file 2 — Additional file 2 PDF tutorial for using ICEKAT. [file 12859_2020_3513_MOESM2_ESM.pdf]

1. Arrange data into colums in Microsoft Excel or other spreadsheet program

Make sure no other extraneous metadata from the plate reader output is included as this will cause errors in ICEKAT

Column A = time

Remaining columns = time-dependent data, separated by condition, and labeled accordingly

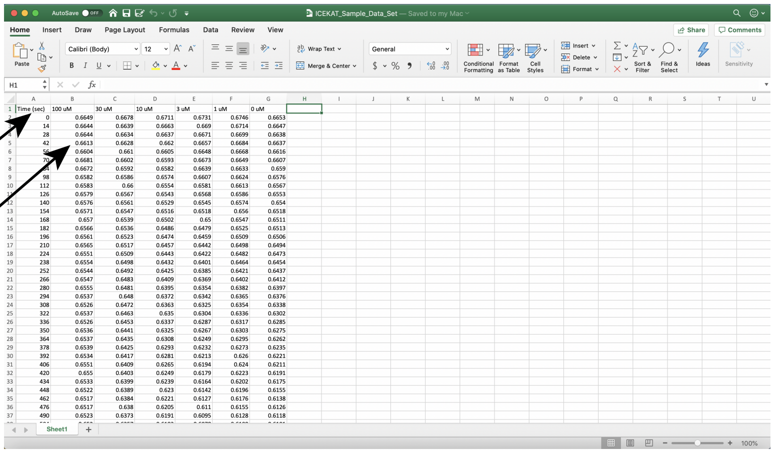

2. Save data in Comma Separated Values (*.csv) format

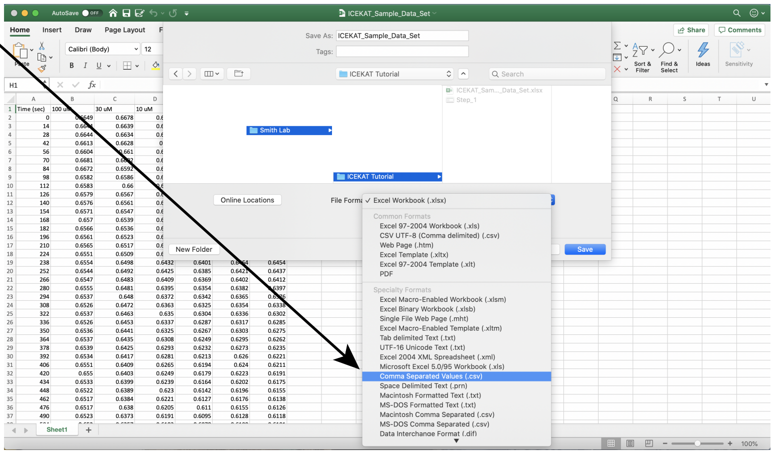

3. Navigate to ICEKAT https://icekat.herokuapp.com/icekat

4. Click ‘Upload Local File’

IC_{50} data is uploaded in this tutorial

5. Select/open the *.csv file containing the data

Displayed data will switch from sample data set to user’s data

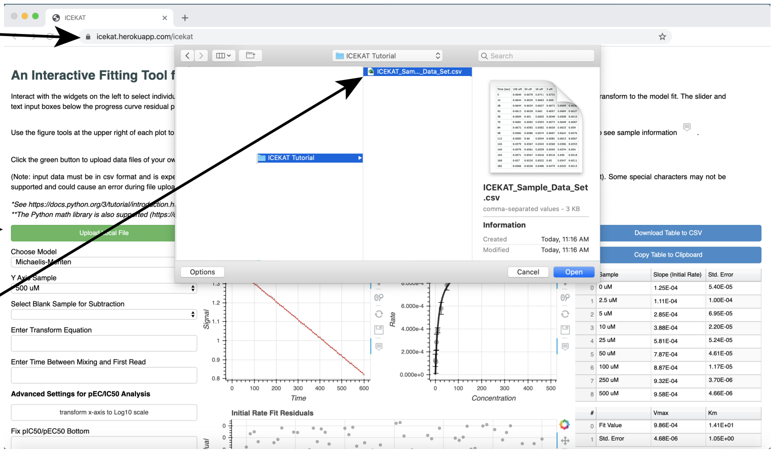

6. Select desired model from the drop down menu

Fitting Model options:

1. Michaelis-Menten

2. pEC50/pIC50

3. High-Throughput Screen

7. Fitting mode can be altered using the toggles above the data display window

Fitting mode options:

1. Maximize Slope Magnitude

2. Linear Fit

2. Logarithmic Fit

3. Schnell-Mendoza (Michaelis-Menten model only)

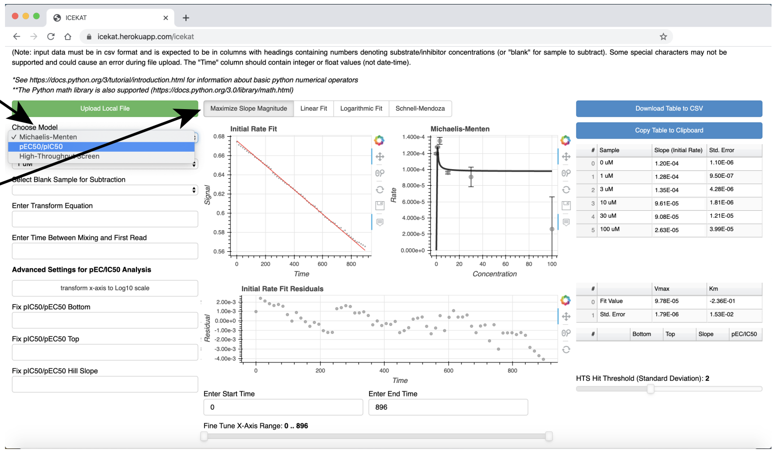

**Default fitting mode is Maximize Slope Magnitude*

8. Automatically displayed values are slopes

To transform into a rate, enter a transform equation

*Type 'x' followed by a transformation equation use ' / ' for division and ' * ' for multiplication*

Displayed values are converted to rates/rate constants

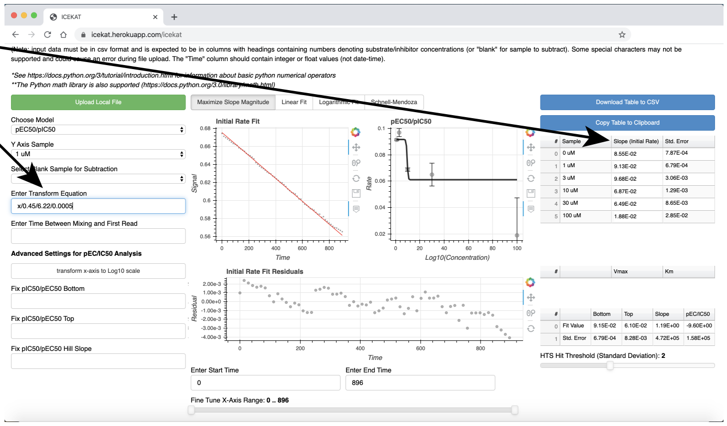

9. Use the "Y Axis Sample" drop-down menu to view the curve for a given condition

The corresponding data will then appear in the 'Initial Rate Fit' box after selection

10. Use the 'Enter Start Time' and 'Enter End Time' boxes and sliders to adjust the portion of the curve being fit, with the goal of selecting the linear portion of the curve and obtaining a random distribution of residuals

Boxes are recommended for course adjustments, and sliders recommended to fine tune the fit

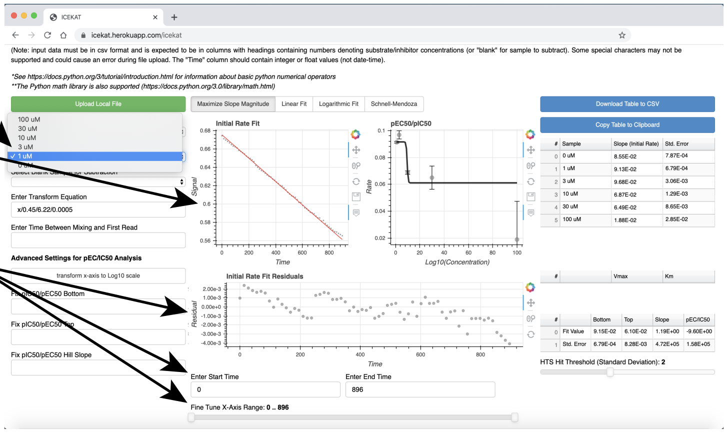

11. If using the 'pEC50/pIC50' model, select 'transform x-axis to Log10 scale'

A fixed Top, bottom, and Hill Slope can be entered to constrain the fit to the pIC_{50}/pEC_{50} data

**Only transform the x-axis if concentrations are uploaded in a linear scale. If concentrations are already in a log_{10} scale, then leave this button unchecked.*

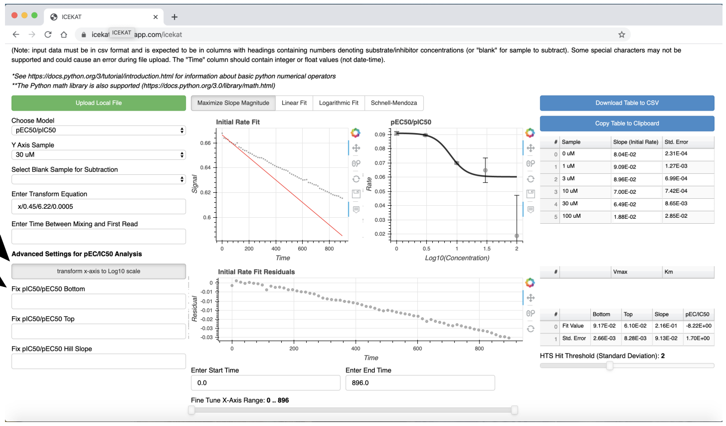

12. Select and adjust the fit for any remaining conditions in the data set

The slope/initial rates for each condition, along with the standard error of the fit and graphical fit are updated in real time.

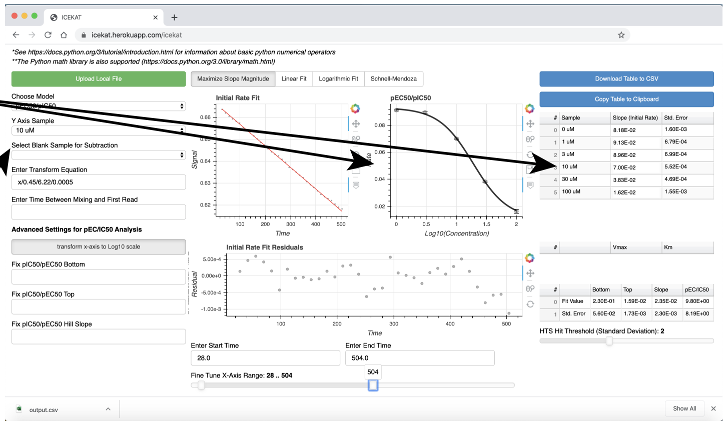

13. A Blank Sample (*e.g.* without added substrate or enzyme) condition may also be selected, and the associated rate subtracted from the rates of the data set

Michaelis-Menten data can be fit using ‘Maximize Slope Magnitude’, ‘Linear Fit’, or ‘Logarithmic Fit’ modes for each individual curve. Alternatively, ‘Schnell-Mendoza’ mode can be used to globally fit an entire substrate titration data set using the Schnell-Mendoza equation.

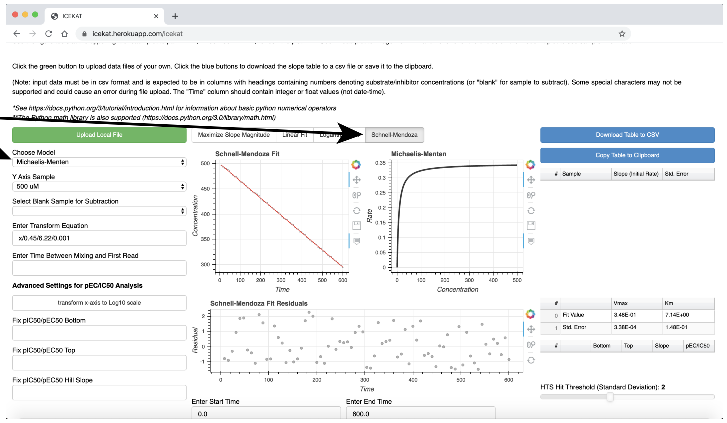

A transform equation MUST be included when fitting data with the Schnell-Mendoza equation as the equation fits the decrease in substrate concentration over time

- *If following an increase in absorbance over time, then the transform equation must be negative (i.e. multiplied by -1)*
- *If the data is already expressed in terms of substrate concentration, then enter ‘x’ in the ‘Enter Transform Equation’ box*

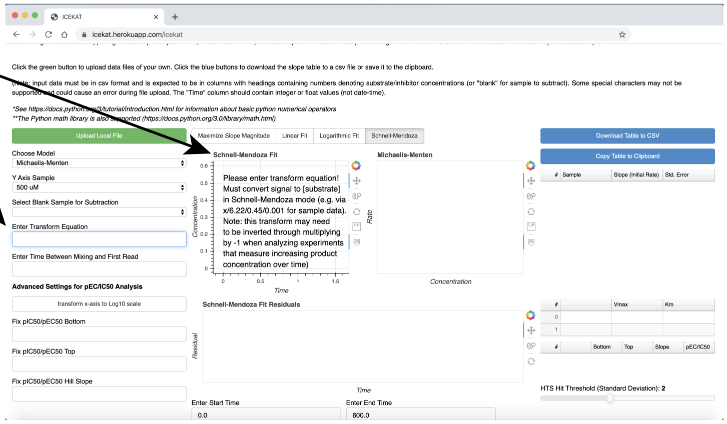

High-throughput screening datasets of potential inhibitors or activators can be analyzed by selecting the ‘High-throughput Screen’ model

- *The ‘High-Throughput Screen’ data output window displays the entire data set, where the black line represents the mean slope/rate*
- *Data points highlighted in red and blue in the output window reflect samples with rates higher and lower than the High Throughput Screening (HTS) Hit Threshold*
- *The number of standard deviations used to determine the HTS Hit Threshold can be adjusted using the slider*

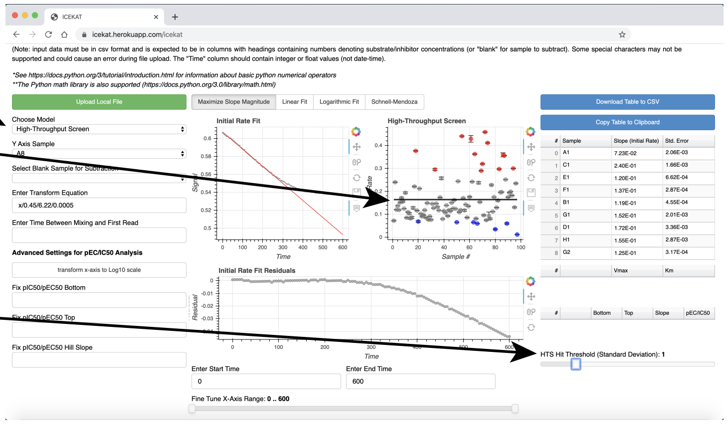

14. When fitting is complete, data may be downloaded or copied using the blue ‘Download Table to CSV’ and ‘Copy Table to Clipboard’ buttons

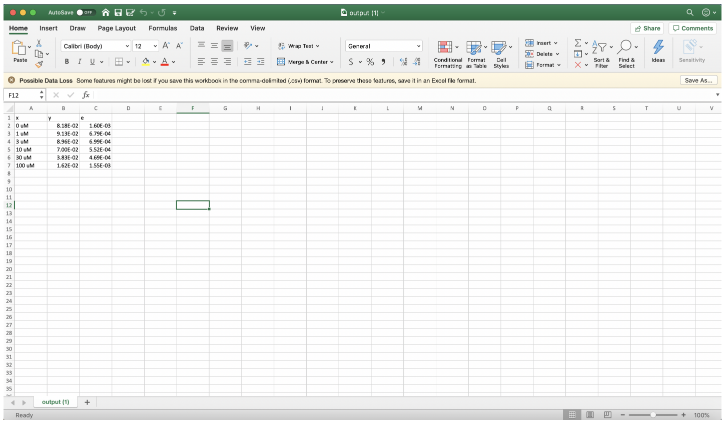

15. The data will export in *.csv format

x (first column) = condition

y (second column) = rate/slope

e (third column) = error of the fit
